# Management of dysphagia in head and neck cancer: current and future perspectives

**DOI:** 10.3389/fonc.2026.1799956

**Published:** 2026-03-20

**Authors:** Charlotte Schellen, Melissa Konings, Alice Vergauwen, Leen Van den Steen, Margot Baudelet, Gwen Van Nuffelen

**Affiliations:** 1 Faculty of Medicine and Health Sciences, Dep. of Translational Neurosciences, University of Antwerp, Antwerp, Belgium; 2 Department of Otolaryngology and Head and Neck Surgery, Rehabilitation Center for Communication Disorders, Antwerp University Hospital, Antwerp, Belgium; 3 Vlaamse Werkgroep Hoofd Hals Tumoren (VWHHT), Ghent, Belgium; 4 Department of Speech and Language Pathology, Thomas More University of Applied Sciences, Antwerp, Belgium; 5 Department of Otolaryngology and Head and Neck Surgery, Ghent University Hospital, Ghent, Belgium; 6 Department of Radiation Oncology, Ghent University Hospital, Ghent, Belgium; 7 Faculty of Medicine and Health Sciences, Dep. of Rehabilitation Sciences, Ghent University, Ghent, Belgium

**Keywords:** (chemo)radiotherapy, dysphagia, fibrosis, head and neck cancer, neuropathy, rehabilitation, surgery, swallowing

## Abstract

Dysphagia is a highly prevalent and disabling complication in patients with head and neck cancer (HNC). Both (chemo)radiotherapy (C)RT and surgical interventions contribute to swallowing dysfunction through mechanisms such as muscle disuse, fibrosis, neuropathy and anatomical alterations. Given its profound impact on nutrition, morbidity and quality of life, effective dysphagia rehabilitation is essential. This narrative review aims to summarize current knowledge on swallowing rehabilitation in HNC and to discuss recent and ongoing clinical trials. Strength-based rehabilitation is well-established, with both prophylactic and reactive interventions demonstrating benefits for muscle strength and swallowing function. However, transfer of these gains to oral intake is suboptimal. Recent research emphasizes the possible benefits of combined approaches integrating strength, functional and skill-based exercises. In surgically treated populations, timing and content of therapy may vary, yet exercise-based protocols generally improve swallowing-related quality of life and oral intake. Swallowing rehabilitation in patients after total laryngectomy remains insufficiently studied, nevertheless evidence indicates that retraining of swallowing-related muscles is feasible. Recent literature reveals several strategies for management of HNC-dysphagia that go beyond strength, skill and functional training with special interest in radiation-induced fibrosis, edema, lower cranial nerve neuropathy, cough reflex and neuromuscular electrical stimulation. Despite the growing body of evidence supporting swallowing rehabilitation in HNC, significant gaps persist regarding optimal intervention content and timing. Ongoing advances in medical treatment, e.g. immunotherapy and radiotherapy de-escalation, necessitate adaptable rehabilitation strategies. Future research should focus on patient-centered approaches and consider the impact of evolving oncological therapies on swallowing outcomes.

## 1 Introduction

Although surgery was initially the only treatment available for head and neck squamous cell carcinoma (HNSCC), (chemo)radiotherapy [(C)RT] and immunotherapy have expanded the treatment landscape for HNSCC over the last two decades (1). Current mainstays of treatment for head and neck cancer (HNC) involve definitive (C)RT or upfront surgery followed by pathology-guided adjuvant (C)RT (2–6). The treatment modality ultimately selected depends on patient- and tumor-related factors, including tumor stage, tumor site, nodal involvement, invasion of adjacent structures and HPV-status (6). Regardless of the treatment modality, the impact on swallowing function can be profound and may significantly affect the biomechanics of swallowing resulting in dysphagia (7–9). HNC-dysphagia is often characterized by decreased muscular strength (e.g. reduced tongue strength, impaired pharyngeal propulsive contraction) and a diminished range of motion (e.g. reduced laryngeal excursion, trismus) of the swallowing musculature (10–15). Consequently, impaired muscle activation may result in residue throughout the mouth and pharynx, thereby affecting swallowing efficiency (16, 17). Furthermore, both treatment and tumor progression may damage nerves involved in swallowing (18). As a result, impaired innervation of the swallowing musculature can disrupt the timing and coordination of the swallowing process (10, 18). This may compromise swallowing safety, as effective swallowing relies on precisely timed airway closure to prevent penetration and aspiration.

Despite continued refinement of RT protocols, including intensity-modulated RT (IMRT) focusing on reducing radiation dose to organs at risk for dysphagia, over 60% of patients develop radiation-associated dysphagia (RAD) (19–23). Within minutes after irradiation an inflammatory process is induced in the submucosa. This triggers a cascade of cellular changes resulting in radiation damage, inhibiting normal cell repopulation needed for regeneration of the tissue (18, 24). RAD may appear as an acute side effect of (C)RT, but it often persists and evolves into a chronic condition due to lymphedema, fibrosis, and muscle atrophy (18, 25–27). These toxicities can arise during treatment and persist long after RT has ended, resulting in chronic RAD (C-RAD) (21, 25). In addition, a delayed onset of RAD has been described, with sixteen percent of patients developing late-onset RAD (L-RAD) defined as RAD occurring five years or more after RT (10, 28, 29).

Although a large proportion of existing research on HNC-dysphagia focuses on patients treated with (C)RT, surgery, whether alone or combined with (C)RT, can likewise result in dysphagia (30). Surgical resection may involve removal of oral, pharyngeal and laryngeal structures essential for swallowing, thereby leading to dysphagia or worsening pre-existing impairments (6, 31). The volume of resection at specific locations, such as the tongue, suprahyoid muscles and pharynx, is a key contributor to postoperative dysphagia (14, 32–36). Closure or reconstruction techniques can also influence postoperative swallowing outcomes (6, 14, 34, 37). Despite favorable oncological outcomes due to advances in surgical techniques like transoral robotic surgery (TORS), transoral laser (micro)surgery (TOLS) or microvascular free flap reconstruction, dysphagia remains prevalent (6, 37). Adjuvant treatment like postoperative RT often exacerbates these problems (14, 31, 32, 34, 37).

HNC-related dysphagia has far-reaching effects on health and daily functioning. It is associated with reduced QoL, malnutrition, dehydration, and aspiration pneumonia and contributes to non-cancer-related mortality in this patient population (20, 34, 38–42). Additionally, patient-reported outcome measures highlight the substantial physical and psychological burden affecting QoL experienced by patients with HNC (43). Therefore, HNC-dysphagia necessitates appropriate care. Traditionally, dysphagia management has been mainly compensatory with dietary restrictions, posture changes and tube feeding (44, 45). Over the past fifteen years however, there has been growing interest in rehabilitative interventions aimed at improving swallowing outcomes (46). Although swallowing rehabilitation remains a relatively young and evolving field, fundamental principles are acknowledged in literature: swallowing therapy should be based on the underlying pathophysiology of dysphagia and grounded in the principles of motor learning, neuroplasticity and exercise (47–50). Further insights based on these principles are expected to advance optimal dysphagia management for HNC patients. One of these key principles is the principle of overload stating that physiologic load during rehabilitation must exceed the natural demand in order to increase muscle strength. This principle is strongly embedded in swallowing exercise protocols targeting muscle strength (47). More recently research on functional and skill-based interventions is gaining increasing attention (51, 52). Both functional and skill-based swallowing therapies are based on the principle of specificity, referring to how closely the exercise task corresponds with the targeted outcome (47). These strategies represent a logical and complementary therapeutic approach to strength training by directly targeting impaired motor planning, coordination, and task-specific swallowing performance.

Research concerning the management of head and neck cancer (HNC)-related dysphagia has mainly focused on prophylactic approaches aimed at preventing radiation-induced dysphagia (RAD), followed by reactive rehabilitation targeting acute C-RAD or L-RAD (10, 53, 54). Recently, however, increasing attention has been directed toward swallowing rehabilitation in surgical populations, including patients undergoing extensive resections of oral, laryngeal, or hypopharyngeal carcinomas (55–58).

The goal of this narrative review is to provide a well-founded overview of published and ongoing trials on strength-, skill-, and function-based swallowing rehabilitation for HNC patients treated with (C)RT and/or surgery. Second, it discusses recent developments in HNC dysphagia management that go beyond strength, functional, and skill training.

## 2 Methods

A systematic search was performed across following databases: Pubmed, Embase, ClinicalTrials.gov (ongoing/active and completed trials), ISCRTN, Cochrane Library. Following search terms were used: ‘dysphagia and head and neck cancer’, ‘skill training and dysphagia’, ‘skill training and head and neck cancer’, ‘therapy or management or exercises or rehabilitation’, ‘head and neck cancer or head and neck cancer neoplasm’, ‘dysphagia or deglutition or deglutition disorders or swallowing’, ‘preoperative or postoperative or operative or surgery or laryngectomy’. The literature was searched for publications from 2010 to January 2026.

## 3 Results

### 3.1 Strength training

Strength-based rehabilitation is one of the most studied, well-acknowledged and widely applied strategies in swallowing management in patients with HNC. Both prophylactic and rehabilitative interventions targeting muscle strength have demonstrated therapeutic benefit (59–64). Fundamental to this approach is the application of the principles of exercise (47). In general, strengthening exercises contain intensive instrumental training of the lips, jaw, tongue, suprahyoid musculature, pharynx constrictors, and cough strength, with some authors incorporating the Mendelsohn maneuver and (super)supraglottic swallow as a rehabilitation technique instead of a compensatory approach (61, 65). Most research combines multiple strengthening exercises in one fixed protocol, although growing evidence suggests that isolated tongue strengthening exercises (66, 67) and cough strength training (68, 69) improve strength and swallow function. However, transfer of increased strength and swallowing towards an ameliorated oral intake in patients with HNC appears to be limited (67) and uncertainty remains regarding the optimal timing and intensity of these interventions.

Ongoing trials, summarized in **Table 1**, are currently investigating several aspects of strength training in HNC patients. These include the effect of prophylactic strength training on oral intake, the influence of different delivery modes of strength training (pen-and-paper versus mobile health and biofeedback–supported interventions) on adherence and self-reported dysphagia and the effect of combining nutritional status monitoring with strength training on swallowing function. Other trials focus specifically on cough strength training and its impact on aspiration and nutritional status.

**Table 1:** Overview of ongoing registered trials concerning swallowing rehabilitation in HNC.

Strength training							
Investigator(s)	Trial title	Identifier	Type of study	Intervention	Timing of intervention	Dosage of intervention	Primary outcome
Lundy, D.S.	Outcomes of Prophylactic Swallowing Therapy in Patients Undergoing Definitive Chemoradiation for Head and Neck Cancer	NCT03435471	Single-site two-arm randomized prospective interventional pilot study	(1) 6 ST sessions with speech-language pathologist (2) 1 ST session with speech-language pathologist prior treatment	Pre-treatment	6 weeks, daily home practice	Change in oral intake
Rieger, J.	Swallowing Therapy With the Assistance of a Mobile Health Device in Head and Neck Cancer Patients: a Pan-Alberta Study	NCT04698499	Two-arm cross-over randomized controlled trial	(1) ST with pen and paper (2) ST with mobile health device and sEMG biofeedback	≥3 months post treatment	3 months, daily total of 8 sets of 3 exercise types with 3 repetitions of each	(1) Percentage adherence to recommended number of exercises (2) Change in self-reported impact of dysphagia
Tuomi, L.	Randomized Study Regarding Preventive Jaw- and Swallowing Intervention for Patients with Head and Neck Cancer (HNC)	NCT04005521	Two-arm randomized controlled trial	(1) ST: Jaw- and swallowing exercise protocol + encouraging patients to eat or drink for as long as possible (2) encouraging patients to eat or drink for as long as possible	Pre- and during treatment	NS	(1) Maximal interincisal jaw opening (2) Swallowing function
Crowder, S.	The ENHANCE Study: Exercise and Nutrition in Head And Neck CancEr Survivors: A Randomized Clinical Trial	NCT05798780	Two-arm randomized controlled trial	(1) ST + nutrition (2) Nutrition	During and post RT	12 weeks, 1 session weekly	Feasibility, adherence and acceptability
Hutcheson, K.	Cough, Expiratory Training, and Chronic Aspiration After Head and Neck Radiotherapy	NCT02662907	Non-randomized trial	ST: Active Expiratory Muscle Strength Training (EMST)	Post treatment	8 weeks, 5 days per week, 5 sets of 5 breaths	Association between aspiration and expiratory function
Pauloski, B.	EMST in Patients Undergoing CRT for HNCA	NCT03916809	Two-arm randomized controlled trial	(1) ST: Active Expiratory Muscle Strength Training (EMST) + Standard Care (2) Sham EMST + Standard Care	Coincident with RT/CRT	8 weeks, 5 days per week, 5 sets of 5 breaths	Feeding-tube-free food intake (days)

Functional and skill-based swallowing therapy							
Investigator(s)	Trial title	Identifier	Type of study	Intervention	Timing of intervention	Dosage of intervention	Primary outcome
Martino et al. (75)	PRO-ACTIVE trial: a randomized study comparing the effectiveness of PROphylACTic swallow InterVEntion for patients receiving radiotherapy for head and neck cancer	NCT03455608	Multi-center 3-arm randomized clinical trial	(1) ST: RE-ACTIVE high intensity intervention (same exercises as (3)) (2) ST + FE: PRO-ACTIVE EAT low intensity prophylactic intervention (3) ST + FE: PRO-ACTIVE EAT+EXERCISE high intensity prophylactic intervention	Depending on type of intervention: RE-ACTIVE arm: as soon as dysphagia is reported PRO-ACTIVE arms: start before RT	Depending on type of intervention: RE-ACTIVE arm: NS PRO-ACTIVE arms: daily practice at home + 2-weekly sessions with SLP	Duration of feeding tube dependency one year post RT
Massonet et al. (79)	HIT−CRAD trial: Home−based intensive treatment of chronic radiation−associated dysphagia in head and neck cancer survivors	ISRCTN57028065	Multi-center 3-arm randomized clinical trial	1) ST (2) ST + FE with HD-tDCS (3) ST + FE with sham HD-tDCS	≥6 months post treatment Chronic RAD present for at least 3 months	1) 8 weeks ST (2) 4 weeks ST + 4 weeks FE with HD-tDCS (3) 4 weeks ST + 4 weeks FE with sham HD-tDCS	Change in functional oral intake
Martin-Harris et al. (92)	Training Swallowing Initiation During Expiration: Impact on Safety and Efficiency Following Treatment for Oropharyngeal Head and Neck Cancer	NCT05278039	Single-center interventional two-arm randomized controlled trial	FE + SKE with biofeedback using a wearable sensor	≥3 months post completion of first-line cancer treatment	6 therapy sessions of +/- 1 hour	(1) Change in frequency (%) of swallows initiated during expiration (2) Change in penetration/aspiration
Schellen et al. (94)	RES-ST: RESpiratory Swallowing Therapy in patients with radiation associated dysphagia	ISRCTN47914607	Single-center interventional two-arm cross-over randomized controlled trial	FE + SKE (respiratory-swallow patterns + super supraglottic swallow) with biofeedback	≥6 months post RT	4 therapy sessions of +/- 1,5 hours in two weeks’ time	Number (%) of patients with reduced penetration/aspiration

Swallowing therapy following surgery							
Investigator(s)	Trial title	Identifier	Type of study	Intervention	Timing of intervention	Dosage of intervention	Primary outcome
Hysjulien, C.	A Comprehensive Approach to Head and Neck Cancer Prehabilitation	NCT04617678	Single-center interventional two-arm non-randomized prospective clinical trial	Prehabilitation: education and interventions, NS	NS	NS	Swallowing-related quality of life
Martino, R. & Irish, J.	ESSI-SURG: Feasibility of Early Swallowing and Speech Intervention for Head and Neck Cancer Patients Treated SURGically	NCT06192771	Single-center interventional two-arm randomized controlled trial	Presurgical education, SLP therapy sessions, NS	Post-operative day 3 and 7, after discharge 4 weekly sessions	4 weekly therapy sessions	Study feasibility
Ku, J.	T-PROSE: Impact of Tongue Proactive Strengthening Exercise Program on Speech and Swallowing Outcomes Following Partial/Hemiglossectomy and Reconstruction	NCT07110142	Single-center interventional two-arm randomized controlled trial	ST: tongue	NS	NS	Quality of life
Chiu, Y.	AASP: App-Assisted Swallowing Program After Oral Cancer Surgery	NCT07301047	Single-center interventional	Swallowing care combined with mobile exercise app, NS	Starting postoperative day 7	NS	Oral dysfunction and oral intake
Coffey, M.	Feasibility of exercises to treat swallowing difficulty after cancer surgery to remove the voice box	ISRCTN95066540	Multicenter interventional randomized unblinded feasibility study	ST: tongue	Within 3 months postoperatively	6 weeks, 4 therapy sessions per week	Swallow function and swallowing-related quality of life

COM, compensatory treatment; FE, functional exercises; ROM, range of motion exercises; SKE, skill-based exercises; ST, strength training.

NS, not specified; LCNP, lower cranial neuropathy.

### 3.2 Functional and skill-based swallowing therapy

In the management of RAD, the principles of “use it or lose it” and “use it and improve it’ (50) represent a key strategy in functional and skill-based rehabilitation (70). Within this approach, the primary focus is not solely to increase muscle strength but to improve overall swallowing function.

#### 3.2.1 Functional swallowing therapy

If improved swallowing is the goal, then swallowing would be the optimal training task. Encouraging patients to continue eating before, during, and after (C)RT can thus be considered as a targeted swallowing exercise aimed at maintaining function and preventing disuse of the swallowing musculature (71). The EAT-RT program (Eat All Through Radiation Therapy), developed by Hutcheson and colleagues, provides a structured framework that supports speech-language pathologists in guiding patients to maintain oral intake throughout treatment (72). It is a proactive, individualized approach that employs a diet hierarchy and structured mealtime routines to encourage patients to continue consuming the most challenging food consistencies they can tolerate.

Even greater benefit appears to be achieved when swallowing exercises are added to an intervention strategy. The combination of maintaining oral intake during treatment and performing strength exercises is associated with improved long-term dietary outcomes after (C)RT, enhanced swallowing safety, and a shorter duration of gastrostomy dependence (71, 73). The PRO-ACTIVE trial compares the PRO-ACTIVE EAT and PRO-ACTIVE EAT + EXERCISE programs in patients undergoing treatment next to a RE-ACTIVE strategy, where intervention starts as soon as dysphagia is detected (74). The hypothesis is that the PRO-ACTIVE therapies will be more effective than the RE-ACTIVE therapy, with an even greater expected benefit in the PRO-ACTIVE approach where eating and exercises are combined.

Another approach is to translate swallowing of food and drinks into an exercise schedule. An example of such a functional exercise based intervention is the McNeill Dysphagia Therapy Program (MDTP) (75). During this intensive, certified three-week program, patients are exposed to progressively challenging swallowing tasks in order to improve swallowing function. Charters and colleagues expanded the original MDTP protocol to a ten-week version and demonstrated its feasibility in HNC patients, along with improvements in swallowing safety and efficiency in a prospective trial (76). In a bootcamp designed swallowing therapy, Hutcheson added strength training to a similar functional exercise program. 81% of the participating patients, improved at least in one domain of swallowing (functional status, penetration/aspiration or swallowing specific QoL (77). The HIT-CRAD trial currently investigates this combined approach by comparing the effects of strength training alone versus a combination of strength training and functional swallowing therapy (78). **Table 1** gives an overview of the ongoing trials in functional oriented swallowing rehabilitation.

Since transfer from increased strength towards oral intake is limited after strength training, there has been growing interest in combined practice approaches that integrate strength-based and functional and/or skill-based training within swallowing rehabilitation. Preliminary, unpublished results from our HIT-CRAD trial suggest that such integrated approaches may be associated with increased oral intake (78). It can therefore be assumed that combination-based interventions may have a greater impact on swallowing function and, consequently, on oral intake. Given the clinical relevance of oral intake as a key functional outcome, this highlights the importance of further exploring (combining) functional and skill-based therapeutic approaches within swallowing rehabilitation.

#### 3.2.2 Skill-based swallowing therapy

Where functional exercises mainly focus on massive repetition of the swallow function while variating in boluses and volume, skill-based swallowing therapy tries to alter this function by targeting specific skills that we (often unconsciously) use and rely on during swallowing (50). Skill-based practice therefore focuses on particular components of swallowing function, such as timing and coordination to enhance both safety and efficiency of swallowing. This is grounded in the concept of neuroplasticity since it involves modification on the level of cortical mechanisms and motor control processes involved in swallowing (48, 51, 79).

Recently, increasing attention goes to a specific timing-related component of swallowing in patients with HNC, namely respiratory–swallow patterns (RSP). In typical RSP, swallowing occurs during an expiration-swallow-expiration pattern (E-S-E) (80). During the expiratory phase, the larynx induces a position that is more favorable for safe swallowing: the tongue is positioned more posteriorly and the pharynx narrows (81, 82). Hyolaryngeal excursion is initiated and the arytenoid–vocal fold complex is slightly adducted. Consequently, initiating a swallow during expiration is considered a protective setpoint that facilitates laryngeal closure and enhances airway protection (81–84). Deviations from this pattern - swallows initiated by inspiration (I-S-E) and/or followed by inspiration (E-S-I, I-S-I) - are classified as pathological RSP (pathRSP) and are associated with reduced airway protection and an increased risk of aspiration and aspiration pneumonia (83, 85). Previous studies report pathRSP in up to 60% of HNC patients following (C)RT (38, 86, 87). Martin-Harris and colleagues conducted a non-randomized study in a heterogeneous HNC cohort demonstrating that skill-based RSP training while using visual feedback has a positive effect on laryngeal vestibular closure, tongue base retraction, pharyngeal residue and penetration and aspiration (88, 89). This research group is currently investigating respiratory–swallowing therapy (RST) in a more diverse HNC population, using advanced software that automatically detects and evaluates RSP performance and provides real-time feedback on performance during therapy (90). Furthermore, they have initiated a randomized, sham-controlled clinical trial in which HNC survivors in an earlier subacute phase of recovery receive RST. To evaluate the feasibility of telehealth and the transfer of learned skills to daily life, this randomized controlled trial uses a wearable sensor linked to an application designed to detect RSP patterns (90–92). Within this context, the RES-ST trial currently investigates the effectiveness of the combination of training RSP through respiratory biofeedback and task-specific, functional swallowing training using the super supraglottic swallow maneuver on airway safety and swallowing efficiency (93). These ongoing trials are presented in **Table 1**.

### 3.3 Swallowing therapy following surgery

Research on swallowing therapy following HNC surgery is commonly divided according to tumor subsite. This review distinguishes two broad, frequently reported and heterogeneous categories: oral cancers and laryngeal/hypopharyngeal cancers. Within the latter group, specific attention is given to patients undergoing total laryngectomy.

#### 3.3.1 Oral carcinomas

Most studies on swallowing therapy and surgical interventions primarily focus on patients with newly diagnosed oral squamous cell carcinoma, however recent attention has shifted toward recurrent disease as well (31, 33, 94–97). Oral carcinomas represent a highly heterogeneous group, with subsites such as the tongue, floor of the mouth, mandible, maxilla, and palate. Despite this heterogeneity, dysphagia appears to be commonplace in the early postoperative phase: FEES assessments performed on postoperative day seven have demonstrated dysphagia in 98% of 400 patients (30).

Within this diverse population, surgical management typically involves tumor resection and subsequent reconstruction, with techniques generally well described at study level. Nevertheless, small sample sizes frequently preclude subgroup analyses regarding the influence of tumor location or surgical approach on swallowing outcomes (31).

Given the complexity and variability of surgical management, increasing attention has been directed toward the timing of swallowing therapy. While some studies describe their approach as ‘prehabilitation’ or ‘proactive’, true preoperative initiation of therapy is rare. To date, only one randomized controlled trial initiates therapy preoperatively, beginning at least one week before surgery with strength and stretch exercises (97). In other studies, ‘proactive’ refers to early initiation after surgery (95, 96). The timing of therapy initiation depends on the patient achieving a medically appropriate condition, which is typically achieved approximately one week postoperatively (33, 94, 95, 98). Most protocols are aiming to start therapy within three weeks after initial resection and reconstruction (31). This variability is also reflected in clinical practice, as evidenced by a survey among speech-language pathologists in New Zealand and Australia, which found no consensus regarding an optimal starting point, with most clinicians initiating therapy between surgery and adjuvant treatment (99).

Despite variability in timing, the content of treatment protocols is relatively consistent across studies. Most interventions include both active and passive oral motor exercises targeting strength and stretch. These typically include range-of-motion exercises targeting the neck, lips, jaw, and tongue (31, 33, 94–97, 100–102), alongside strength training focused primarily on the tongue and pharyngeal musculature (31, 95, 97, 102, 103). In addition to motor exercises, sensory stimulation is also commonly employed, using techniques such as cold acid stimulation, thermal stimulation, vibration or air pulse stimulation (33, 94, 101, 102). Some protocols also include swallowing maneuvers such as the Mendelsohn maneuver, effortful swallow, and supraglottic swallow or protective strategies as effective coughing (95, 96, 98, 101–103). While most studies describe fixed exercise schedules combining various techniques, Zhang and colleagues provide personalized exercise programs based on the results of clinical swallowing examination (98).

Although the central focus of intervention is exercise-based therapy, patient education (e.g. information about oral hygiene and compensatory techniques) is also routinely provided (94, 98). Additionally, patients are encouraged to maintain oral intake, even in combination with a nasogastric tube (95, 96).

Considerable heterogeneity is observed in prescribed dose of therapy. Most intervention protocols prescribe daily exercises, typically two to three sessions per day, although one study advises five daily sessions (31, 94–98, 100–104). Session duration generally ranges from 20 to 30 minutes (94–96, 103). Only one study explicitly applies the principle of overload, reporting training intensity in terms of 60–80% of the one-repetition maximum (103).

The total duration of training varies considerably, from brief interventions lasting 10–14 days to programs extending 8–12 weeks (98, 102). The need for further intervention may be based on clinical swallowing examination (94).

The implementation of these exercise-based protocols has been associated with improvements in swallowing-related QoL, swallowing safety and efficiency, and functional oral intake. Swallowing-related QoL is often severely impaired in the immediate postoperative period due to pain and discomfort, however several studies report a gradual recovery over time following intervention (33, 94, 95, 98, 102, 105). Nevertheless, the long-term effects remain insufficiently understood, as a marked decline in swallowing-related QoL has been reported at two years post-treatment in some studies (95, 96). Swallowing function, as assessed by clinical examination and instrumental evaluations, demonstrates improvements in both safety and efficiency (31, 94–98, 101, 103). These functional changes are mirrored by recovery in oral intake. Although oral intake is significantly disrupted immediately after surgery, recovery is reported after one month, with the majority of patients no longer requiring tube feeding three months postoperatively (94–97). Importantly, treatment adherence appears to play a critical role, with higher adherence associated with better functional oral intake outcomes (94–96, 104).

Ongoing clinical trials, summarized in **Table 1**, are expected to further strengthen the evidence base, by evaluating early, systematic speech-pathology interventions, as well as the effectiveness of comprehensive multidisciplinary prehabilitation (106, 107). Additionally, attention is being paid to the integration of home-based, device-assisted exercises and the potential role of mobile application-supported swallowing care (108, 109).

#### 3.3.2 Laryngeal/hypopharyngeal carcinomas

Total laryngectomy plays an important role in the management of laryngeal and hypopharyngeal carcinomas, either as primary treatment for advanced-stage disease or as salvage surgery for tumor recurrence or severe swallowing dysfunction following (C)RT. Despite the profound anatomical and functional consequences of this procedure, this subgroup has received minimal attention with regard to dysphagia (6, 110). As a result, the incidence of swallowing impairment in this population is underestimated (111). Self-reported prevalence rates of post-laryngectomy dysphagia reach up to 72%, with a substantial negative impact on QoL (41, 111–113).

The pathophysiology of post-laryngectomy dysphagia is multifactorial. Swallowing outcomes are influenced by the pharyngeal closure technique, the extent of additional pharyngeal mucosal resection, postoperative complications, and the use of adjuvant (C)RT (34, 37, 114). Overall, swallowing outcomes appear to be poorer following flap reconstruction compared with primary closure (37). However, consensus regarding the optimal surgical closure technique for swallowing outcomes is lacking, largely due to substantial methodological heterogeneity across studies (34).

Beyond surgical technique, resection-related anatomical and functional changes contribute to swallowing impairment. Reduced pharyngeal contraction may compromise bolus transport and neopharyngeal efficiency. The absence of hyolaryngeal elevation eliminates mechanical stretch, potentially resulting in hypopharyngeal pooling. In addition, increased resistance at the pharyngoesophageal junction may cause functional obstruction (15). Postoperative complications, including strictures and pseudovalleculae formation, can be underlying causes of swallowing problems (34, 37, 110, 114, 115). Importantly, in cases of new-onset dysphagia following total laryngectomy, tumor recurrence must always be excluded (114). Both primary and postoperative RT negatively affect swallowing function (116), with CRT having the most profound negative impact (37).

Traditionally, swallowing therapy aims to preserve or restore adequate oral intake while ensuring airway protection. However, airway protection is no longer a rehabilitation concern following total laryngectomy (117). In recent years, increasing attention has been directed toward the development and evaluation of dysphagia-specific rehabilitation strategies for individuals after total laryngectomy. Initially, it was unclear whether the residual musculature post-laryngectomy could be activated through swallowing exercises. However, recent research has demonstrated that both conventional and resistance-based exercises are capable of eliciting muscle activation in this population (118).

Most reported interventions prescribe daily exercise practice over periods ranging from six to twelve weeks (117, 119–121). In contrast, training intensity remains poorly defined, with only one study explicitly reporting adjustments in exercise resistance (117).

The content of the therapeutic interventions primarily targets mobility and muscle strength. Mobility-focused components include flexibility exercises for the head, neck, and shoulders, as well as range-of-motion exercises for the tongue, lips, and jaw. When facial lymphedema is present, additional targeted exercises are incorporated (119). Strength training mainly focuses on the tongue, jaw, and suprahyoid musculature (117, 120, 121). Exercises incorporating adjustable resistance are associated with the highest percentage of muscle activation compared to conventional approaches (118). In addition to exercise therapy, several interventions emphasize patient education and self-care strategies for TL (119, 122, 123).

Implementation of these protocols has been associated with improvements in swallowing function, specific strength measures, functional oral intake and swallowing related QoL. Gains in swallowing function are supported by both patient-reported outcomes and instrumental assessments (57, 117, 119, 121). Neijman and colleagues re-evaluated treatment effects following an eight-week detraining period and again at six months post detraining period. Although some decline of effects was observed over time, outcomes consistently remained superior to baseline levels (57, 117).

To further support the broader implementation of these protocols, a preliminary trial (**Table 1**) is currently underway as a precursor to a randomized controlled trial, examining the feasibility of specialized training for speech-language pathologists, the acceptability of the prescribed exercises, and the cost-effectiveness of the intervention (124).

### 3.4 Beyond strength, functional and skill training

#### 3.4.1 Radiation-induced fibrosis and lymphedema

Radiation-induced fibrosis and lymphedema are key factors underlying C-RAD and L-RAD. Approximately 69% of HNC patients are dealing with external lymphedema nine months post-treatment, whereas 47% of HNC patients develop moderate to severe fibrosis within 12 months of RT (125, 126). Head and neck lymphedema can cause inflammation, fibrosis and reduced mobility of swallowing related structures (126). Fibrosis on the other hand is thought to stiffen connective tissues, reduce capacity for contraction and appropriate range of motion and compress peripheral nerve tracts, thereby contributing to diminished strength, flexibility, and in some cases denervation of swallowing muscles (127). Supportive and medical interventions that can prevent or limit the development of fibrosis and lymphedema are therefore highly relevant.

Recently, the MANTLE nonrandomized clinical trial investigated the feasibility, safety and functional outcomes of manual therapy (MT) in survivors of HNC with fibrosis-related late RAD (127). The MANTLE program targets cervical range of motion and deeper swallowing regions-of-interest, including the tongue, pharynx and larynx, through soft tissue mobilization and cervical stretching/strengthening (128). MT was found to be safe and feasible and was associated with several functional, physical, and psychosocial gains, including maximal incisor opening, cervical range of motion and lymphedema-fibrosis symptom severity. However, there was a minimal effect on swallowing outcome measures.

Currently there is little consensus and strong practice variation in head and neck lymphedema management (129). Two recent reviews acknowledge the positive effects multi-component therapy programs, often referred to as complete decongestive therapy (CDT), on external lymphedema (130, 131). CDT focuses on compression, manual lymphatic drainage, exercises and skin care (132).

International experts have different views on the exact content of the CDT, but there is a relative consensus on the need to include exercises that improve head and neck mobility (e.g., range of motion and flexibility) and exercises that improve swallowing (129). Preliminary data on the effectivity of swallowing exercises in laryngectomized patients are derived from a clinical phase II study in which 20 participants completed a six-weeks, resistance-based swallowing rehabilitation program (57, 117). Patient reported prevalence of chin oedema, based on a binary yes/no-question, reduced from 70% at baseline to 20% post swallowing rehabilitation. Further evidence is needed to objectify the suggestion that swallowing exercises improve chin oedema. Of note, a pilot study on self-care and the use of pneumatic compression garments showed that patients reported improvements in their swallowing function (133).

#### 3.4.2 Timing of swallowing rehabilitation in lower cranial nerve neuropathy

Radiation fibrosis is thought to compress peripheral nerve tracts, thereby contributing to denervation of critical swallowing muscles. Recent literature identifies lower cranial neuropathy (LCNP) as a significant contributor to late radiation-associated dysphagia (134, 135). The lower cranial nerves that are most vulnerable to treatment are the glossopharyngeal IX, vagus X and the hypoglossal XII, with the latter being the mostly reported (134, 136–139).

The presence of LCNP presumably negatively affects patients’ potential for swallowing rehabilitation. From that perspective, the time period between treatment and the onset of LCNP potentially offers a unique window of opportunity to address RT-induced biomechanics and preserve the patients’ swallowing function. In other words, swallowing rehabilitation in HNC-patients may be time-dependent (128). This idea is supported by the results of previous outcome studies (77, 128, 140). A secondary analysis of a large, multi-center swallowing therapy trial among 117 survivors with chronic and late RAD, showed that QoL and diet scores improved most among those who started therapy <1 year after RT, whereas little improvement was evident among those who started therapy more than 2 years post-RT (128, 141). Conversely, a secondary analysis of the large HIT-CRAD-trial found that patients who were more than 5 years post radiotherapy showed significant improvements in swallowing-related outcomes after 8 weeks of swallowing rehabilitation. These improvements were even comparable to those of participants who were less than 5 years post radiotherapy (25).

Of interest regarding the management of LCNP is the ongoing ‘Stop LCNP’- phase I/II trial that investigates the side effects and best dose of steroid therapy (prednisone or methylprednisolone) for improving symptoms of late radiation-associated LCNP in oropharyngeal cancer survivors. Preliminary results showed that high-dose steroid therapy was well tolerated. Oral/per-gastrostomy prednisone at 3mg/kg dosing met criteria to move to phase II testing for the indication of symptomatic radiation associated cranial nerve XII neuropathy (142).

#### 3.4.3 Cough reflex

Since cough strength and effectiveness are increasingly recognized as key components of airway protection in the presence of dysphagia, there is a growing interest in the assessment of cough function and reflex across different patient populations, including HNC patients (143–146). Beyond assessment, the training of cough function has gained attention as a potential rehabilitation strategy and interventions targeting the cough reflex are being explored. Sensory-based reflex training, for example through the inhalation of cough-inducing agents, aims to stimulate laryngo-pharyngeal sensory pathways and train a more effective and coordinated cough response (147–149). Such approaches may be particularly relevant in HNC patients due to their sensory impairments following (C)RT (150–152).

#### 3.4.4 Neuromuscular electrical stimulation

Transcutaneous neuromuscular electrical stimulation (TNMES), involving application of a low-voltage current to the neck skin via surface electrodes in order to strengthen the oropharyngeal musculature, was already described ten years ago showing contrary conclusions (140, 153, 154). A more recent randomized trial in 120 patients with nasopharyngeal carcinoma, describes the proactive use of TNMES targeting the suprahyoid and infrahyoid musculature, the hypoglossal nerves, and the deeper pharyngeal constrictor muscles. They showed significant improvement in pharyngeal swallowing compared to exercise based training and comparable increases in QoL for both types of swallow training (155).

#### 3.4.5 Immunotherapy

In the ongoing pursuit of treatment strategies to improve overall survival in HNC, immunotherapy has emerged as promising modality. Immunotherapy is characterized by low toxicity and high specificity, often administered in combination with other treatment modalities (156). For example, immunochemotherapy has been proposed as a promising approach to reduce the extent of surgical resection, thereby potentially facilitating improved functional recovery (157).

Despite this growing body of evidence, the effects of immunotherapy on swallowing function remain insufficiently explored in the current literature. Swallowing outcomes are seldom incorporated as explicit endpoints, as most studies prioritize oncological measures such as tumor response and survival (157). In some studies, dysphagia is already recognized as one of the most common or severe serious adverse reactions (158, 159). A number of ongoing trials have begun to incorporate swallowing function and swallowing-related quality of life as outcome measures (160–162). Other studies include swallowing as a secondary endpoint or indirectly through subscales of broader instruments (163).

#### 3.4.6 Dose de-escalation in RT

To reduce the side effects of (chemo)radiotherapy, increasing attention is being given to radiotherapy de-escalation strategies that preserve oncologic outcomes (164). Current research explores the potential of elective neck irradiation (ENI), focusing on both reductions in irradiated volume and administered dose (165). Furthermore, (chemo)radiotherapy appears to be more effective in patients with HPV-positive head and neck cancer, making de-escalation a viable option in this subgroup (166). Advances in surgical techniques, such as transoral surgery, also offer opportunities for postoperative de-escalation, which may lead to improved swallowing outcomes (167). A recent study evaluated de-escalation strategies in HPV-associated patients primary treated with TORS, followed by postoperative RT. Patients receiving reduced-dose RT demonstrated significantly better swallowing outcomes. However, this benefit was no longer observed at 2 years of follow-up (167). These ongoing improvements in medical treatment imply that the underlying pathophysiology of swallowing disorders may differ over time.

## 4 General conclusion

Research on dysphagia rehabilitation in patients with HNC continues to expand. Several ongoing clinical trials are investigating different rehabilitation approaches as well as the optimal timing of intervention. This review of the current and upcoming literature highlights several notable aspects of current research in this domain.

Earlier research clearly showed the positive effects of specific types of strength training on muscle strength and swallowing (59, 61, 62). More recent studies, however, have increasingly investigated combinations of muscle strength training with functional and skill-based rehabilitation (74, 78). This latter approach may be particularly valuable because (C)RT has demonstrated effects on both strength loss and functional decline. Each of these therapeutical strategies induces specific changes in the biomechanics of swallowing. When combined, these mechanisms may synergistically improve swallowing function. Further high-quality research is needed to clarify the added value and optimal integration of functional and strength training components within swallowing rehabilitation for HNC patients treated with (C)RT. However, to understand the specific changes in biomechanics induced by exercise, there is also a need for high-level studies examining the effect of a single exercise modality.

In contrast to (C)RT, surgery is not an organ-preserving approach. To optimize functional recovery, reported swallowing therapy in this group predominantly focuses on strength-based exercises (56). Although, heterogeneity in tumor subsites, surgical procedures and intervention protocols limits conclusions regarding the optimal timing, content, and intensity of rehabilitation. While short-term benefits of swallowing therapy following surgery are consistently reported, evidence regarding long-term outcomes remains limited (55, 56). This is partly due to the frequent use of adjuvant RT, which substantially affects swallowing function (55, 94, 98). Future studies should therefore explicitly integrate the influence of RT in their design and analysis.

In patients undergoing total laryngectomy, swallowing rehabilitation has received relatively little attention, although interest in this area is increasing (6, 34). Emerging evidence demonstrates that swallowing-related muscles can be retrained despite significant anatomical alterations (57, 117, 118). More standardized research is needed to establish evidence-based rehabilitation protocols for this patient population (57).

Approaches that address radiation-induced fibrosis and lymphedema in HNC patients are emerging and represent important, potentially modifiable targets for swallowing intervention. Although current evidence remains limited, preliminary data suggest that manual therapy and multi-component approaches such as complete decongestive therapy are feasible, and beneficial for physical and symptom-related outcomes (128, 130–132). Their direct impact on swallowing function however remains modest and warrants further investigation. In parallel, radiation-induced lower cranial neuropathy has been identified as a critical contributor to L-RAD, highlighting the importance of early recognition and timely initiation of swallowing rehabilitation (134, 135). The concept of time-dependent responsiveness to therapy further supports the existence of a potential window of opportunity during which functional decline may be attenuated before irreversible neural compromise occurs (128). Within this context, pharmacological strategies, including high-dose steroid therapy for radiation-associated lower cranial neuropathy, are emerging as promising adjuncts that may complement rehabilitative interventions (142).

Although research and evidence in HNC populations remains limited, cough reflex assessment and training represent a promising addition to swallowing rehabilitation. Further research is needed to determine optimal protocols and clinical effectiveness in this population (150–152). Likewise, NMES continues to show mixed but evolving evidence, with recent trials suggesting potential advantages over exercise-based therapy in selected populations (140, 153, 155).

As immunotherapy becomes increasingly integrated into HNC care, systematic evaluation of its effects on swallowing function is essential to fully understand its functional implications and to guide future rehabilitation strategies (157–159). Finally, dose de-escalation strategies continue to be explored with the aim of maximizing functional preservation. Ongoing developments in this field may also influence swallowing function, which could shift the patient population requiring rehabilitation (167). It is highly important to collect data on swallowing-related outcome variables in future studies on these new medical strategies, to ensure that swallowing therapy can be adapted to the actual needs of HNC patients with dysphagia.

In conclusion, the scientific evidence supporting the benefits of swallowing therapy for HNC-related dysphagia is growing. There is a clear, scientifically based foundation for strength, skill, and/or function-based swallowing therapy for HNC-dysphagia, whether it is used prophylactically or for rehabilitation. Furthermore, strategies that go beyond these therapeutic approaches are emerging and offer promising prospects for the future.
